# Dental caries in association with viral load in children living with HIV in Phnom Penh, Cambodia: a cross‐sectional study

**DOI:** 10.1186/s12903-021-01441-2

**Published:** 2021-03-25

**Authors:** Kimiyo Kikuchi, Junko Yasuoka, Sovannary Tuot, Sumiyo Okawa, Sokunthea Yem, Pheak Chhoun, Makoto Murayama, Chantheany Huot, Siyan Yi

**Affiliations:** 1 Department of Health Sciences, Faculty of Medical Sciences, Kyushu University, Fukuoka, Japan; 2 Research and Education Center for Prevention of Global Infectious Diseases of Animals, Tokyo University of Agriculture and Technology, Tokyo, Japan; 3 KHANA Center for Population Health Research, Phnom Penh, Cambodia; 4 Department of Community and Global Health, Graduate School of Medicine, The University of Tokyo, Tokyo, Japan; 5 Cancer Control Center, Osaka International Cancer Institute, Osaka, Japan; 6 Kawasaki City Dentists Association, Kanagawa, Japan; 7 National Paediatric Hospital, Phnom Penh, Cambodia; 8 Center for Global Health Research, Touro University California, Vallejo, CA USA; 9 Saw Swee Hock School of Public Health, National University of Singapore and National University Health System, Singapore, Singapore; 10 School of Public Health, National Institute of Public Health, Phnom Penh, Cambodia

**Keywords:** Acquired immunodeficiency syndrome, Cambodia, Cross‐sectional study, HIV, Dental caries, Viral load

## Abstract

**Background:**

Oral health status is associated with the overall health among people living with human immunodeficiency virus (HIV) infection. However, it is unclear whether dental caries is associated with the viral load in this population. Particularly, dental caries among children living with HIV needs better understanding as this can affect their overall health and future well-being. This study assessed the association between dental caries and viral load among children living with HIV in Phnom Penh, Cambodia.

**Methods:**

This cross-sectional study, conducted at the National Pediatric Hospital as a baseline survey of a randomized controlled trial, included 328, 3–15-year-old children living with HIV and their primary caregivers. Calibrated and trained examiners conducted oral examinations for dental caries (DMFT/dmft index) in the children and retrieved the latest HIV viral load data from the hospital’s patient information system. On the dental examination day, the children and their caregivers were invited to answer a questionnaire-based interview. Multiple logistic regression analysis was conducted to assess the association between dental caries and viral load. The cut-off point for undetectable viral load was set at < 40 copies/mL.

**Results:**

Data from 328 children were included in the analysis; 68.3% had an undetectable viral load. The mean DMFT/dmft was 7.7 (standard deviation = 5.0). Adjusted regression analysis showed that dental caries in permanent or deciduous teeth was positively associated with detectable viral load (adjusted odds ratio [AOR]: 1.07, 95% confidence interval [CI]: 1.01–1.14). Conversely, antiretroviral therapy of ≥ 1 year and self-reported better adherence to antiretroviral drugs were negatively associated with detectable viral load. Among children with detectable viral load, dental caries in permanent or deciduous teeth was positively associated with non-suppression of viral load (> 1000 copies/mL) (AOR: 1.12, CI: 1.03–1.23).

**Conclusions:**

Dental caries was associated with viral load status detection among children living with HIV. This finding suggests that dental caries may affect their immune status. The oral health of children living with HIV should be strengthened, and further research is needed to clarify the causal relationship between viral load and oral health status.

## Background

Globally, acquired immunodeficiency syndrome (AIDS)-related deaths decreased by 35%, from 1.7 million in 2004 (the highest rate) to 1.1 million in 2018 [1]. This change implies that people living with human immunodeficiency virus (HIV) now have a longer life expectancy than previously reported [2] and, as a result, the number of people living with HIV is higher than ever [1].

Cambodia is one of the Southeast-Asian countries that has implemented effective HIV prevention and treatment approaches [3]. In 2018, there was a 62% reduction in new HIV infection cases among the general population of all ages, including children, compared to 2010 [1]. Of all people living with HIV, 81% received antiretroviral therapy (ART) and 78% had suppressed viral load in 2018 [1]. However, a high prevalence of dental caries has been reported among uninfected Cambodian children; approximately 93% of children aged 6 years and 80% aged 12–13 years had dental caries [4, 5]. Children living with HIV are no exception to poor oral health, and the condition is even worse than in uninfected children. Thus, these children have a higher number of dental caries than children without HIV infection [6].

The presence of oral lesions indicates HIV progression and an increase in the plasma viral load, which is the amount of HIV in the blood of people living with HIV [7–9]. Among these patients, oral hairy leukoplakia and oral candidiasis are suggested to be associated with a decreased viral load [10, 11]. A HIV-seropositive status could be associated with dental caries due to progressive immunodeficiency [12], influence of medications on salivary flow [13], diet, inadequate oral hygiene, and low socioeconomic status [14]. However, the association between HIV viral load and dental caries remains unclear, especially in the pediatric population. Childhood is a crucial period in which physical development determines the future oral health status and, consequently, may influence overall health.

This study therefore aimed to assess the association between dental caries and viral load among children living with HIV in Phnom Penh, Cambodia.

## Methods

### Study design

This cross-sectional study was conducted from February to April 2018 as a baseline survey of a randomized controlled trial (Clinical Trial Number: ISRCTN15177479) aiming to improve the oral health of children living with HIV at the National Pediatric Hospital, Phnom Penh, Cambodia. Details of the trial have been published elsewhere [15]. This hospital is a tertiary referral facility that provides comprehensive care and treatment for children from across the country, including HIV and dental services, and is a major pediatric HIV clinic in Phnom Penh that provides ART. Children living with HIV receive consultation and collect their medication every 2 months. Dental care is provided free of charge to children living with HIV in this hospital.

### Study population

The target population in this study were children living with HIV and their caregivers. Children who visited the hospital and self-administered their medication were interviewed without the need for their caregivers. The children were included if they were aged 3–15 years on the day of the data collection, had a patient identification number at the study site hospital, and were receiving ART. They were selected from the hospital’s ART clinic registry using an age-stratified random sampling method. Randomization was performed using a computerized algorithm by a data analyst who was not a primary member of the research team. The caregivers were eligible only if they were ≥ 18 years old and were the primary caregiver of the child.

### Sample size

The sample size for the children was calculated based on the number required for the following intervention phase. The sample size set in the study protocol was calculated according to the decayed, missing, or filled permanent teeth (DMFT) score obtained from a previous survey among 8–15-year-old children living with HIV [15]. However, after the completion of the baseline survey among the 3–15-year-old children, we obtained accurate DMFT scores for the study population. Therefore, we revised the sample size based on the following indicators: increment in DMFT, 17%; baseline DMFT of children living with HIV, 4.0 (standard deviation [SD] = 3.6); power, 80%; alpha, 5%. The final sample size required was 199 for each group. However, because of the improvement in preventing mother-to-child transmission in Cambodia, the number of children living with HIV aged < 8 years was low, and we could not recruit the required sample size. Therefore, 160 children were recruited in each group. In this study, both intervention and control groups of children living with HIV were examined, and thus, in total, the required sample size was 320 children.

### Data collection

Two teams, each consisting of one dentist and one dental assistant, collected data on the children’s dental caries status. To ensure accuracy of the examination, one of the researchers (a dentist) provided a one-day training session on assessing the dental status of patients using the World Health Organization guidelines [16]. The reproducibility of intra-examiner and inter-examiner evaluations was assessed. The dentists checked for DMFT in 10 children and compared the results between the two groups. The consistency rate of the results was ≥ 0.85. These data were not included in the main data collected. The total number of DMFT was calculated as the DMFT score, and the decayed, missing, or filled deciduous teeth (dmft) score was obtained for deciduous teeth. The overall DMFT and dmft values were evaluated separately and together by the sum of both scores. The severity of dental caries was expressed based on DMFT/dmft = 0 (no dental caries) and DMFT/dmft > 0 (the presence of dental caries). If permanent and deciduous teeth were found to occupy the same tooth space, the status of the permanent tooth was recorded according to the World Health Organization guidelines [16]. All dental data were collected in the dental unit of the hospital using disposable mouth mirror.

The research assistants retrieved clinical data from the HIV clinic’s registered database, including age, latest viral load within 12 months, ART regimen, and duration of ART. Six research assistants interviewed the caregivers and children on the same day of dental data collection using a structured questionnaire developed based on previously published questionnaires, including the child’s adherence to antiretroviral drugs [17, 18]. They received one-day training from the first author to clarify and improve their understanding of the questionnaire. For the question on adherence, if the drugs were self-administered by the child, we interviewed the child to obtain accurate information. The question was, “How would you rate your/your child’s adherence over the past 30 days?” The response choices were very poor, poor, fair, good, very good, or excellent [17].

### Statistical analyses

The data were analyzed descriptively to assess the distribution of the variables. Subsequently, dental caries was classified into dental caries in either the permanent teeth, deciduous teeth, or in all the teeth. We assessed the association of viral load, the dependent variable, with dental caries, age, sex, duration of ART, and adherence to antiretroviral drugs. The age, sex, and duration of ART variables were those included in a model used in a previous study that examined the association between DMFT and CD4 + cell count [12]. The adherence to antiretroviral drugs variable was also included because it is related to viral load in most cases [19]. For all the participants, the independent variables were first examined for association with viral load (“detected” or “undetected”), defined using a cut-off point of < 40 copies/mL according to the detection limit of the tests. We also examined association with viral non-suppression, 1000 copies/mL, which is the threshold for treatment failure [20] among only those who had a detectable viral load. For bivariate analyses, we used the Chi-square test or Fisher’s exact test, if a count in one cell was < 5, for categorical variables. We used Student’s *t*-tests for continuous variables. Further, we applied the multiple logistic regression analysis, and *p* < 0.05 was used to indicate statistical significance. All data analyses were performed using IBM SPSS Statistics for Windows, version 24.0 (IBM Corp., Armonk, NY, USA).

## Results

In total, 337 children living with HIV participated in our study. However, data from nine children were excluded owing to missing values. Finally, data from 328 children were included in the analysis. Among the interview responses, 100 were children-only responses while 228 were from both children and caregivers.

### General characteristics

Table 1 shows the characteristics of participating children. Of 328 children, 48.2% were female. The mean age of the children was 10.8 years (standard deviation [SD] = 3.0). The mean duration of ART was 6.3 years (SD = 3.6). Moreover, 68.3% of children had an undetectable viral load. Among children with a detectable viral load, the load ranged up to approximately 5,012,000 copies/mL. Among the participants, 93.0% had at least one permanent tooth (age range, 6–15 years old); 57.3% had at least one deciduous tooth (age range, 3–15 years old). Of them, 50.3% had both permanent and deciduous teeth (age range, 6–15 years old). The mean values for DMFT and dmft were 4.0 (SD = 3.6) and 7.0 (SD = 4.9), respectively. The mean number of teeth with caries including permanent and deciduous teeth was 7.7 (SD = 5.0). Only 5.8% of children were free from caries. For adherence to antiretroviral drugs, no children answered, “very poor.” The most frequent response was “good” (51.2%).

**Table 1:** General characteristics and health status of the study participants

Characteristic		All children (n = 328)	Children with detectable viral load (n = 104)
Sex, n (%)			
Female		158 (48.2)	46 (44.2)
Male		170 (51.8)	58 (55.8)
Age (years), mean (SD)		10.8 (3.0)	10.3 (3.3)
3–5		19 (5.8)	9 (8.7)
6–7		31 (9.5)	15 (14.4)
8–10		78 (23.8)	20 (19.2)
11–12		91 (27.7)	27 (26.0)
13–15		109 (33.2)	33 (31.7)
Duration of ART (years), mean (SD)		6.3 (3.6)	4.9 (3.7)
< 1		28 (8.5)	19 (18.3)
≥ 1		300 (91.5)	85 (81.7)
Viral load, n (%)			
Undetected		224 (68.3)	–
Detected (> 40 copies/mL)		104 (31.7)	–
Viral load detected (n = 104) (copies/mL), n (%)			
< 40		–	26 (25.0)
40–100		–	16 (15.4)
100–1000		–	12 (11.5)
1000–10,000		–	12 (11.5)
10,000–100,000		–	28 (26.9)
> 100,000		–	10 (9.6)
Children without dental caries, n (%)		19 (5.8)	5 (4.8)
Dental caries status, n (%)			
DMFT (total children, n = 305) (children with detectable viral load, n = 93)		4.0 (3.6)	4.2 (4.1)
dmft (total children, n = 188) (children with detectable viral load, n = 68)		7.0 (4.9)	7.8 (5.0)
DMFT/dmft (total children, n = 328) (children with detectable viral load, n = 104)		7.7 (5.0)	8.9 (5.3)
Adherence to antiretroviral drugs in the past 30 days, n (%)			
Very poor		0 (0.0)	0 (0.0)
Poor		3 (0.9)	3 (2.9)
Fair		25 (7.6)	9 (8.7)
Good		168 (51.2)	54 (51.9)
Very good		75 (22.9)	28 (26.9)
Excellent		52 (15.9)	8 (7.7)
Unknown		5 (1.5)	2 (2.0)

*SD* standard deviation, *ART* antiretroviral therapy, *DMFT* decayed, missing, or filled permanent teeth, *dmft* decayed, missing, or filled deciduous teeth

### Association between dental caries and detectable viral load

Table 2 shows the unadjusted and adjusted regression models on the associations between different factors and a detectable viral load. The adjusted analyses indicated that the total DMFT/dmft was positively associated with a detectable viral load (odd ratio [OR]: 1.07, 95% confidence interval [CI]: 1.02–1.12). Being on ART for ≥ 1 year (OR: 0.19, 95% CI: 0.08–0.43) and “excellent” adherence to antiretroviral drugs (OR: 0.24, 95% CI: 0.08–0.70) were significantly associated with an undetectable viral load.

**Table 2:** Association between dental caries and viral detection (> 40 copies/mL)

Variable	Unadjested analysis^a^			Adjusted analysis								
				Viral non-suppression status among children who had permanent teeth (n = 305)			Viral non-suppression status among children who had deciduous teeth (n = 188)			Viral non-suppression status among all children (n = 328)		
	Unadjusted OR^b^	95% CI^c^		Adjusted OR	95% CI		Adjusted OR	95% CI		Adjusted OR	95% CI	
Dental caries status												
DMFT^d^	1.02	0.96	1.10	1.02	0.94	1.10	–	–	–	–	–	–
dmft^e^	1.06	0.99	1.12	–	–	–	1.10	1.01	1.19*	–	–	–
DMFT/dmft	1.07	1.02	1.12**	–	–	–	–	–	–	1.07	1.01	1.14*
Sex												
Female	Ref.	–	–	Ref.	–	–	Ref.	–	–	Ref.	–	–
Male	1.26	0.79	2.01	1.35	0.80	2.37	1.67	0.86	3.24	1.39	0.84	2.29
Age (years)^f^												
3–10	Ref.	–	–	Ref.	–	–	Ref.	–	–	Ref.	–	–
11–15	0.80	0.50	1.29	0.91	0.49	1.66	2.11	0.94	4.77	1.03	0.59	1.82
Duration of ART^g^ (years)^h^												
< 1	Ref.	–	–	Ref.	–	–	Ref.	–	–	Ref.	–	–
≥ 1	0.19	0.08	0.43**	0.18	0.07	0.45**	0.19	0.07	0.57**	0.22	0.09	0.52*
Adherence to antiretroviral drugs in the past 30 days												
Poor/fair	Ref.	–	–	Ref.	–	–	Ref.	–	–	Ref.	–	–
Good	0.63	0.28	1.43	0.51	0.22	1.18	0.79	0.21	3.02	0.56	0.24	1.31
Very good	0.79	0.33	1.92	0.64	0.26	1.61	0.83	0.20	3.43	0.63	0.25	1.58
Excellent	0.24	0.08	0.70*	0.21	0.07	0.65**	0.21	0.04	0.97*	0.16	0.05	0.51**

^a^Viral load was a binary variable (detected or undetected)

^b^OR, odds ratio

^c^CI, confidence interval

^d^DMFT, decayed, missing, or filled permanent teeth

^e^dmft, decayed, missing, or filled deciduous teeth

^f^The cut-off was set as 10 years old (median of mixed dentition)

^g^ART, antiretroviral therapy

^h^The cut-off was set as 1-year (most likely period attaining viral RNA thresholds in low- and middle-income countries [32])

*p < 0.05; **p < 0.01

Adjusted regression analyses were performed in three models stratified by the presence of certain teeth: permanent teeth only, deciduous teeth only, and any teeth. The model that included children with permanent teeth (n = 305) showed that ≥ 1 year on ART (adjusted OR [AOR]: 0.18, 95% CI: 0.07–0.45) and “excellent” adherence to antiretroviral drugs (AOR: 0.21, 95% CI: 0.07–0.65) were negatively associated with a detectable viral load. The model that included children with deciduous teeth (n = 188) showed that dmft was positively associated with detectable viral load (AOR: 1.10, 95% CI: 1.01–1.19). At least 1 year on ART (AOR: 0.19, 95% CI: 0.07–0.57) while “excellent” adherence to antiretroviral drugs (AOR: 0.21, 95% CI: 0.04–0.97) were negatively associated with detectable viral load. The model that included children with all types of teeth (n = 328) showed that total DMFT/dmft was positively associated with a detectable viral load (AOR: 1.07, 95% CI: 1.01–1.14), and that ≥ 1 year on ART (AOR: 0.22, 95% CI: 0.09–0.52) and “excellent” adherence to antiretroviral drugs (AOR: 0.16, 95% CI: 0.05–0.51) were negatively associated with a detectable viral load.

### Association between dental caries and viral non‐suppression

Table 3 shows the association between dental caries and viral non-suppression (> 1000 copies/mL). Unadjusted analyses showed that dmft (OR: 1.12, 95% CI: 1.01–1.25) and total DMFT/dmft (OR: 1.10, 95% CI: 1.02–1.19) were positively associated with viral non-suppression.

**Table 3:** Association between dental caries and viral non-suppression (> 1000 copies/mL)

Variable	Unadjusted analysis^a^			Adjusted analysis								
				Viral non-suppression status among children who had permanent teeth (n = 93)			Viral non-suppression status among children who had deciduous teeth (n = 68)			Viral non-suppression status among all children (n = 104)		
	Adjusted OR^b^	95% CI^c^		Adjusted OR	95% CI		Adjusted OR	95% CI		Adjusted OR	95% CI	
Dental caries status												
DMFT^d^	0.99	0.90	1.10	1.00	0.90	1.12	–	–	–	–	–	–
dmft^e^	1.12	1.01	1.25*	–	–	–	1.22	1.05	1.41*	–	–	–
DMFT/dmft	1.10	1.02	1.19*	–	–	–	–	–	–	1.12	1.03	1.23*
Sex												
Female	Ref.	–	–	Ref.	–	–	Ref.	–	–	Ref.	–	–
Male	1.92	0.87	4.20	1.50	0.63	3.55	1.22	0.39	3.81	1.87	0.81	4.34
Age (years)^f^												
3–10	Ref.	–	–	Ref.	–	–	Ref.	–	–	Ref.	–	–
11–15	0.88	0.40	1.91	1.00	0.38	2.65	3.88	0.97	15.4	1.49	0.59	3.78
Duration of ART^g^ (years)^h^												
< 1	Ref.	–	–	Ref.	–	–	Ref.	–	–	Ref.	–	–
≥ 1	0.80	0.30	2.17	0.67	0.22	2.10	0.71	0.18	2.71	0.83	0.28	2.52
Adherence to antiretroviral drugs in the past 30 days												
Poor/fair	Ref.	–	–	Ref.	–	–	Ref.	–	–	Ref.	–	–
Good	0.86	0.25	3.01	0.83	0.23	2.97	0.69	0.08	6.25	0.75	0.20	2.86
Very good	1.00	0.26	3.87	0.75	0.18	3.12	1.76	0.17	18.8	0.92	0.22	3.96
Excellent	1.00	0.17	5.99	1.14	0.16	8.17	0.47	0.03	6.84	0.68	0.10	4.91

^a^Viral load was a binary variable with a cut-off of log3.00 (= 1000 copies/mL)

^b^OR, odds ratio

^c^CI, confidence interval

^d^DMFT, decayed, missing, or filled permanent teeth

^e^dmft, decayed, missing, or filled deciduous teeth

^f^The cut-off was set as 10 years old (median of mixed dentition)

^g^ART, antiretroviral therapy

^h^The cut-off was set as 1-year (most likely period attaining viral RNA thresholds in low- and middle-income countries [32])

*p < 0.05; **p < 0.01

Among children with a detectable viral load who had deciduous teeth (n = 68), dmft was positively associated with viral non-suppression (AOR: 1.22, 95% CI: 1.05–1.41). Among all the children with a detectable viral load (n = 104), the analyses indicated that total DMFT/dmft was positively associated with viral non-suppression (AOR: 1.12, 95% CI: 1.03–1.23).

## Discussion

In this study, we found that dental caries, duration of ART, and adherence to antiretroviral drugs were associated with viral load. To the best of our knowledge, this is the first study to reveal the association between dental caries and viral load among children living with HIV.

The undetectable viral load rate of 68.3% in our study was similar to that reported in previous studies [21, 22]. In an intervention study that aimed to examine the effectiveness of ART among Cambodian children living with HIV, the undetectable viral load rate was 81% [22]. A meta-analysis that included seven studies on children living with HIV from 2010, later estimated the undetectable viral load rate (< 40 copies/mL) as 72.7% (95% CI: 62.6–82.8) after 6 months on first-line ART [21].

In our study, the high occurrence of dental caries in permanent or deciduous teeth was associated with a detectable viral load. Furthermore, a high number of dental caries in permanent or deciduous teeth was associated with viral non-suppression. However, this finding contrasted with a study in the United States conducted by Moscicki et al. [23] who found no association between viral load and oral health indicators among children and adolescents aged 7–16 years. However, most of the participants in that study had permanent teeth, and their results do not completely contradict those from our study. We also did not find an association when we included only children with permanent teeth. However, the reason for the lack of this association is unclear. To address this, children’s immature immunological status or the dental risk factors during development will have to be studied. For adults living with HIV, some studies suggested an association between dental caries and advanced viral load. In a study in the United States, Baqui et al. [7] demonstrated that the majority of adults with HIV with a high viral load had a DMFT score > 20. However, the correlation was not significant. Some studies, including our previous study that focused only on children 8–15-year-old, demonstrated a positive association between dental caries and CD4 + cell count among children living with HIV [12, 24]. This may suggest a correlation between dental caries and viral load, as CD4 + cell count has a negative correlation with viral load. With respect to the association between viral load and dental caries, people living with HIV in the advanced stages of AIDS experience xerostomia. It is often diagnosed due to a lack of salivary flow among people living with HIV in the advanced stages of immunosuppression [25]. The lack of salivary flow could be one of the causes of dental caries [25, 26]. However, the causal relationship is not clear, and the influence of poor oral health status on AIDS-related diseases cannot be ruled out based on our results. Alternatively, the compromised systemic health of children may be a factor associated with low immune status, higher viral load, and poor oral health, although further study is needed to clarify the mechanism. However, considering the association between general health and oral health status, more efforts and resources need to be directed toward education and clinical care of children’s teeth.

Our study also demonstrates that other factors, such as duration of ART and adherence to antiretroviral drugs, were associated with viral load. Similar to our research, longer duration on ART and high adherence to antiretroviral drugs were found to be associated with viral suppression or lower viral load in different studies [21, 27–30]. This association implies the effectiveness of ART in improving the immune system. In many cases, the self-reported adherence rate is often biased due to improper recalling and social desirability [31]. However, the positive association identified in our study may suggest that participants’ responses are close to reality.

This study has some limitations. A cross-sectional design was employed, and there was no control group. Therefore, this study does not prove causality. We assessed the presence of dental caries and need further studies to assess other dental risk factors. In addition, as the data were collected from only one hospital, it may not be generalizable. However, our study site hospital includes the largest number of children living with HIV in the country and is the main referral hospital for children living with HIV in Phnom Penh. Thus, our study findings are applicable to the general population of children living with HIV in Cambodia.

## Conclusions

In conclusion, this study revealed a significant association between HIV viral load and dental caries among children living with HIV in Phnom Penh. Thus, the oral health status might have affected the disease progression, as well as the immune status of the children. Further research is needed to clarify the causal relationship between viral load and the oral health status.

## Data Availability

The datasets used and/or analyzed during the current study are available from the corresponding author on reasonable request.

## Abbreviations

AIDS: acquired immunodeficiency syndrome
AOR: Adjusted OR
ART: Antiretroviral therapy
DMFT: Decayed, missing, or filled permanent teeth
dmft: Decayed, missing, or filled deciduous teeth
HIV: Human immunodeficiency virus
OR: Odds ratio
SD: Standard deviation
